# Mental Health and Addictions in Pregnancy: Feasibility and Acceptability of a Computerized Clinical Pathway and Prevalence Rates

**DOI:** 10.1192/j.eurpsy.2024.183

**Published:** 2024-08-27

**Authors:** R. Carmona Camacho, J. Chamorro Delmo, M. Alvaro Navidad, N. Lopez Carpintero, N. Estrella Sierra, R. Guimaraes de Oliveira, M. Olhaberry Huber, L. Mata Iturralde, R. Álvarez García, E. Baca Garcia

**Affiliations:** ^1^Psychiatry; ^2^Obstetrics and ginecology, Jimenez Diaz Foundation; ^3^Obstetrics and ginecology, Tajo University Hospital; ^4^Psychiatry, Infanta Elena University Hospital, Madrid, Spain; ^5^Child and Adolescent Psychiatry, Hassenfeld Children’s Hospital at NYU Langone, New York, United States; ^6^Psychology, Pontificia Universidad Católica de Chile, Santiago, Chile; ^7^Psychiatry, Rey Juan Carlos University Hospital, Madrid, Spain

## Abstract

**Introduction:**

Mental Health problems and substance misuse during pregnancy constitute a serious social problem due to high maternal-fetal morbidity (Cook et al, 2017; JOCG, 39(10) ,906-915) and low detection and treatment rates (Carmona et al. Adicciones. 2022;34(4):299-308)

**Objectives:**

Our study aimed to develop and test the feasibility and acceptability of a screening and treatment clinical pathway in pregnancy, based on the combination of e-Health tools with in-person interventions and, secondly, describe the prevalence of mental illness and substance use problems in this population.

**Methods:**

1382 pregnant women undergoing her first pregnancy visit were included in a tailored clinical pathway and sent a telematic (App) autoapplied questionnaire with an extensive battery of measures (WHO (Five) Well-Being [WHO-5],Patient Health Questionnaire [PHQ-9], General Anxiety Disorder [GAD-7], Alcohol Use Disorders Identification Test [AUDIT], Drug Abuse Screening Test [DAST], Columbia Suicide Severity Rating Scale [C-SSRS] and specifically designed questions on self-harm and psychopharmacological drugs).

Patients who did not respond to the questionnaire on their own received a counseling call.

Based on the screening results, patients were classified into five groups according to severity (Figure 1) and assigned a specific action pathway (Figure 2) that included a range of intervention intensity that goes from an individual psychiatric appointment to no intervention.

**Results:**

Of the 1382 women included in the clinical pathway, 565(41%) completed the evaluation questionnaires. Of these, 205 (36%) were screened as positive (Grades III,IV or V. Table 1) and 3(0.5%) were classified as needing urgent care. Of the patients offered on-line groups (100), 40% (40) were enrolled in them.

**Table 1:** Grade distribution of those screened as positives

Grade III	97 (17,2%)
Grade IV	105 (18,6%)
Grade V	3 (0,5%)

Concerning prevalence rates, 73 (12,9%) patients endorsed at least moderate anxiety according to GAD-7 (≥10), 65 (11,5%) endorsed at least moderate depression according to PHQ-9 (≥ 10), 17 were positive on DAST (3%) and 63 (11%) patients scored above the threshold in AUDIT-C(≥ 3) for alcohol use.

**Image:**

**Figure:**
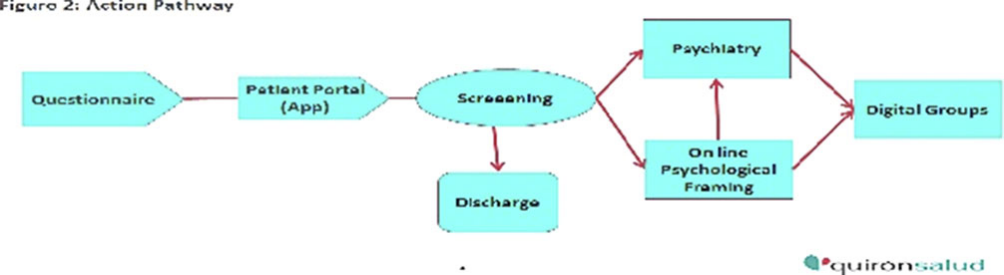

**Image 2:**

**Figure:**
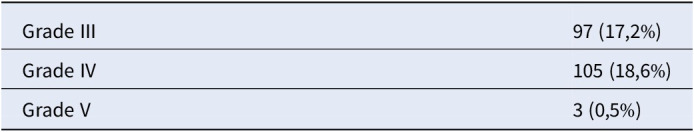

**Conclusions:**

High prevalence rates suggest that effective detection and treatment mechanisms should be integrated into usual care. The use of standardized clinical pathways can help with this aim, allowing better clinical management and referral to treatment, but still face challengues to increase retention. The use of e-health tools offers the opportunity to improve accessibility and therapeutic outcomes through online interventions.

**Disclosure of Interest:**

None Declared

